# Transferred monolayer MoS_2_ onto GaN for heterostructure photoanode: Toward stable and efficient photoelectrochemical water splitting

**DOI:** 10.1038/s41598-019-56807-y

**Published:** 2019-12-27

**Authors:** Mostafa Afifi Hassan, Min-Woo Kim, Muhammad Ali Johar, Aadil Waseem, Min-Ki Kwon, Sang-Wan Ryu

**Affiliations:** 1 Department of Physics, Chonnam National University, Gwangju, 61186 Republic of Korea; 2 Department of Photonic Engineering, Chosun University, Gwangju, 61452 Republic of Korea; 3 Optoelectronics Convergence Research Center, Chonnam National University, Gwangju, 61186 Republic of Korea

**Keywords:** Nanoscale materials, Photocatalysis

## Abstract

Solar-driven photoelectrochemical water splitting (PEC-WS) using semiconductor photoelectrodes is considered a promising solution for sustainable, renewable, clean, safe and alternative energy sources such as hydrogen. Here, we report the synthesis and characterization of a novel heterostructure MoS_2_/GaN to be used as a photoanode for PEC-WS. The heterostructure was synthesized by metal-organic chemical vapor deposition of single crystalline GaN onto a c-plane sapphire substrate, followed by the deposition of a visible light responding MoS_2_ monolayer (E_g_ = 1.9 eV) formed by a Mo-sulfurization technique. Our experimental results reveal that MoS_2_/GaN photoanode achieved efficient light harvesting with photocurrent density of 5.2 mA cm^**−2**^ at 0 V *vs* Ag/AgCl, which is 2.6 times higher than pristine GaN. Interestingly, MoS_2_/GaN exhibited a significantly enhanced applied-bias-photon-to-current conversion efficiency of 0.91%, whereas reference GaN yielded an efficiency of 0.32%. The superior PEC performance of the MoS_2_/GaN photoelectrode is mainly related to the enhanced light absorption due to excellent photocatalytic behavior of MoS_2_, which reduces charge transfer resistance between the semiconductor and electrolyte interface, and the improvement of charge separation and transport. This result gives a new perspective on the importance of MoS_2_ as a cocatalyst coated onto GaN to synthesize photoelectrodes for efficient solar energy conversion devices.

## Introduction

With the concern of a global energy crisis and subsequent desire for alternative fuel sources, hydrogen (H_2_) has drawn considerable interest as a future energy carrier since it is a clean fuel that produces no harmful byproducts after combustion^1,2^. H_2_, in particular, is one of the most promising energy carriers and fuel sources due to its high energy-per-mass content, wide range of available storage and transport approaches, and reduced harmful emissions^3,4^. Indeed, the energy stored into its chemical bond can be used in fuel cells to produce clean electricity. One of the most promising solutions is represented by the use of photoelectrochemical cells (PECs) for light-driven water splitting. PEC water splitting using solar energy has attracted considerable attention in both scientific and industrial communities^5^. Because the fabrication process is simple and environmentally friendly, it has been considered one of the most attractive ways to produce H_2_^6^. After it was firstly demonstrated by Fujishima and Honda in 1972 using TiO_2_ to split water into H_2_ and O_2_, researchers have been studying candidates of high performance photoelectrode for PEC water splitting^7–9^. To achieve high energy conversion efficiency, photoanodes should effectively harvest the sunlight and facilitate charge transfer while they should exhibit long-term stability^10^. The conversion efficiency of solar energy to chemical energy in hydrogen molecule and the stability during PEC process are crucial features of PEC cells, which are mainly determined by the semiconducting material properties of the photoelectrodes^11,12^. Therefore, many semiconductor materials including transition metal oxides have been explored primarily for PEC cells, such as TiO_2_^13^, ZnO^14^, BiVO_4_^15^, α-Fe_2_O_3_^16^ and WO_3_^17^ due to the chemical stability, low cost, and easy fabrication^18^. However, they generally have wide band gaps and absorb a limited portion of the solar spectrum, resulting in poor conversion efficiencies^19^. In pursuit of high-efficiency photoelectrodes for solar energy conversion applications, several crucial requirements should be satisfied, such as a suitable band gap value and appropriate band alignment of the working electrode with the water redox levels^20^. In recent decades, tremendous efforts have been devoted toward exploring novel and efficient photoelectrode materials to improve the solar energy photoconversion efficiency. In order to gain desired solar-to-hydrogen conversion efficiency, heterojunction structures are desirable and considered as an important strategy because they allow for the combination of properties from each element, leading to an improved overall efficiency^21^. The fabricated heterojunction not only can expand the spectral range for light-absorption, but also can promote photoexcited electron–hole separation, which can minimize electron–hole recombination, thus significantly enhancing the energy efficiency^22^.

Recently, researchers have studied III-nitride based materials as potential candidates for PEC water-splitting and the generation of hydrogen energy due to their tunable bandgap, which spans nearly the entire solar spectrum, and a band-edge potential that meets the required water redox potentials^23,24^. Among such III-nitride semiconductor materials used for achieving a desirable solar-to-hydrogen conversion efficiency, GaN has been intensively investigated due to its amazing intrinsic properties and nanotechnological importance. GaN has straddling band-edge positions with the redox potentials of water, which is a prerequisite for efficient charge transfer between a semiconductor and water-based electrolyte^19^. Its wide band gap can be tuned from ultraviolet to near infrared by forming alloy mixture with AlN and InN, which is useful for improving optical and structural properties^25^. In addition, GaN is an inexpensive, non-toxic material with excellent chemical stability against harsh PEC process^26^. As a result of the advantages mentioned above, GaN-based photoanodes have attracted scientists’ attention as a competitive material for harvesting solar energy. However, a desired configuration of a highly efficient and reliable solar hydrogen generation system is still limited due to the disadvantages of the current status of GaN based photoanodes. Thus, several requirements should be satisfied such as a reliable and stable photoanode against photocorrosion and enhanced conversion efficiencies. The use of heterojunction structures or nanostructured cocatalysts with GaN photoanodes is an important strategy to achieve improved PEC performance^27^. The cocatalyst roles not only can protect the photoanode against corrosion, but also may reduce the carrier recombination loss, thus significantly increasing the efficiency of the water splitting process. For instance, the deposition of NiO cocatalyst on GaN reported by our group was a good technique to inhibit surface corrosion by mitigating hole accumulation at the GaN/electrolyte surface^28,29^.

Recently, two-dimensional (2D) transition-metal-dichalcogenides (TMDCs) have intrigued physicists and material scientists due to their distinctive optical and electrical properties, which can be beneficial for numerous devices and applications^30–32^. Among various 2D TMDCs, the molybdenum disulfide (MoS_2_) is especially attractive because of its tunable optical band gap, high chemical stability, earth abundance, nontoxicity, and low cost in comparison with noble metals^33,34^. In-plane closely packed hexagonal arrangements of S–Mo–S atoms with covalent bonds compose two dimensional MoS_2_ structure^35^. These layers are held together by relatively weak van der Waals forces, which provide an advantage for exfoliating MoS_2_ film into monolayers^36^. Intrinsic MoS_2_ offers n-type semiconducting properties, with a tunable band gap (E_g_ = 1.2–1.9 eV) that is classified by the number of layers^37^. Bulk MoS_2_ has an indirect band gap of Eg = 1.2 eV, while monolayer MoS_2_ (ML-MoS_2_) has a direct band gap of E_g_ = 1.9 eV^37,38^, which suggests promise for application in PEC hydrogen generation^39^. Very recently, ML-MoS_2_ was considered as an important catalyst for both photocatalytic and electrocatalytic H_2_ evolution reactions due to the existence of abundant exposed edges, with active sites stemming from the sulfur edges of the MoS_2_ crystal layers^40,41^. Moreover, MoS_2_ thin films or nanosheets have been deposited onto ZnO NWs as cocatalysts, which suppressed the photocorrosion and increased the photocurrent densities^42^. Yong-Jun *et al*. reported that the intimate and large contact interface between MoS_2_ and TiO_2_ can efficiently promote photoinduced charge carrier separation, which leads to an extension of the charge carrier lifetime against recombination^43^.

Herein, we report the synthesis and characterization of patterned highly crystalline ML-MoS_2_ on GaN by a Mo-sulfurization technique. We also demonstrated application of the MoS_2_/GaN heterostructure as a photoanode for PEC water splitting and proposed a mechanism for the process. The PEC characteristics are investigated in detail to evaluate the water splitting behavior of the heterostructure. In addition, improved PEC water splitting performance of the transferred ML-MoS_2_ onto GaN photoanode enabled by the photocatalytic behavior of ML-MoS_2_ is studied. The outcome of the study is that the MoS_2_/GaN photoanode shows a better enhanced PEC performance than pristine GaN. Therefore, the present study can be useful for the development of heterostructure based photoanodes in PEC applications.

## Results and Discussion

**Figure 1:**
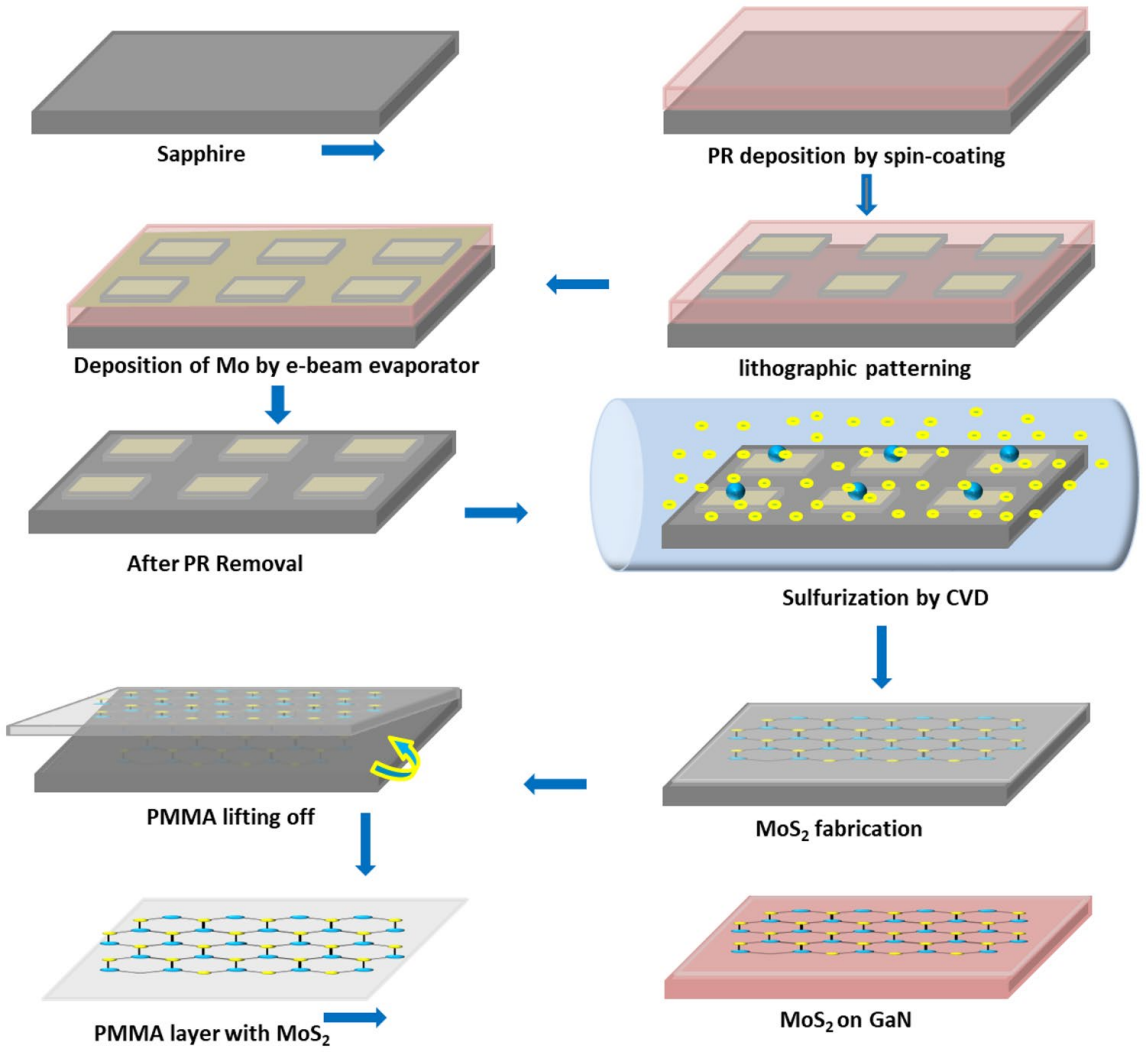
Schematic representation for the growth and transfer process of MoS_2_ monolayer arrays onto a GaN substrate to fabricate a MoS_2_/GaN heterostructure photoanode.

**Figure 2:**
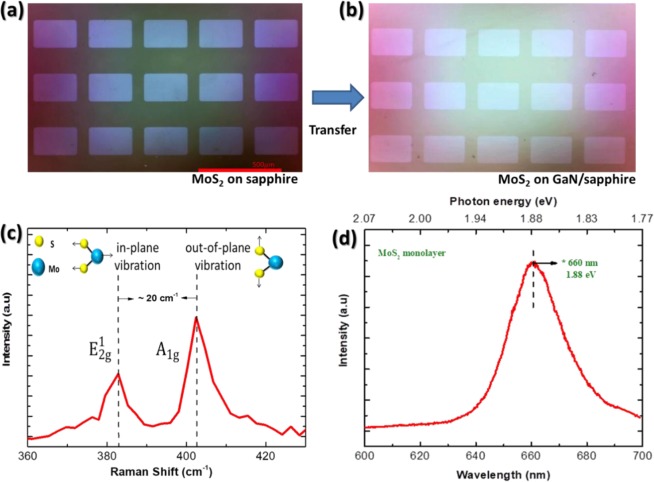
(**a,b**) Optical microscope images of a large continuous film of the synthesized monolayer MoS_2_ with rectangular shaped patterns before and after the transfer process placed onto the sapphire and GaN substrates, respectively. (**c**) Typical Raman spectra for the monolayer MoS_2_ grown on sapphire substrates. (**d**) Room-temperature PL spectra for the monolayer MoS_2_ obtained using a 532-nm laser as an excitation source.

A schematic diagram of the MoS_2_ synthesis procedure and the fabrication of MoS_2_/GaN heterostructure photoanode is displayed in Fig. 1. Initially, a clean 2-inch sapphire substrate was patterned by photolithography to form square lattice patterns. A 1 nm-thick film of Mo was deposited on the sapphire substrate by using e-beam evaporation. The patterned Mo thin films were sulfurized into MoS_2_ in a furnace, then the MoS_2_ film was transferred to GaN on sapphire. Figure 2(a,b) shows the optical microscope images of the monolayer MoS_2_ (ML-MoS_2_) before and after the transfer process placed onto the sapphire and GaN substrates, respectively. It can be seen that the ML-MoS_2_ maintains the same morphology after transfer. It is observed that this transfer technique does not introduce any cracks in the sample which suggests a clean surface and high-quality for the as-transferred samples. Patterned substrates with a uniform distribution of rectangular ML-MoS_2_ (approximately 190 × 110 µm^2^ in size and 50 µm apart) were obtained. Further systematic characterizations of the synthesized ML-MoS_2_ were conducted with Raman spectroscopy and photoluminescence (PL) measurements. Raman spectroscopy is a common tool for determining the precise number of layers in MoS_2_ films such as tri, bi, and ML-MoS_2_. The Raman spectra for these MoS_2_ films contain peaks due to the ${{\rm{E}}}_{2{\rm{g}}}^{1}$ and ${{\rm{A}}}_{{\rm{g}}}^{1}$ phonon modes. The spectra displayed in Fig. 2(c) showed two typical Raman modes for the as-synthesized MoS_2_, ${{\rm{E}}}_{2{\rm{g}}}^{1}$ at 382.71 cm^−1^ and ${{\rm{A}}}_{{\rm{g}}}^{1}$ at 402.61 cm^−1 ^^44,45^. The former corresponds to the opposite vibration of two sulfur atoms in regard to the molybdenum (Mo) atom in the basal plane (in-plane vibration mode), while the latter results from the vertical vibration of only sulfur (S) atoms in opposite directions (out-of-plane vibration mode), as illustrated in the insets of Fig. 2(c)^46^. As is known, the frequency difference ($\Delta {\rm{u}}$) between these two Raman modes is quite sensitive to the layer thickness, and can thus be used to detect the number of MoS_2_ two dimensional layers^47–49^. The ${\,{\rm{E}}}_{2{\rm{g}}}^{1}$ to ${{\rm{A}}}_{{\rm{g}}}^{1}$ peak position difference ($\Delta {\rm{u}}$) in the Raman spectra shown in Fig. 2(c) is ∼20  m^−1^ which can be considered as strong evidence for ML-MoS_2_. The aforementioned results match with those found in previous studies^45,50^. To investigate the optical properties of the prepared ML-MoS_2_, room-temperature PL spectra were collected over a range of 600–700 nm using a 532-nm laser as an excitation source. Figure 2(d) depicts the PL spectra measured for the ML-MoS_2_ grown on a sapphire substrate. A strong PL peak (radiative recombination of A excitons) centered at 660 nm without a shoulder peak at ∼610  nm (optical transitions from B excitons) was observed. In ML-MoS_2_, the excited electrons and holes recombine through direct band-to-band transition at the K-point in the Brillouin zone^37^, leading to strong PL near 1.88 eV. The superior optical properties and the observation of a single peak (A excitonic emission) again represent strong evidence that the ML-MoS_2_ synthesized here is undoubtedly of high quality. Moreover, X-ray diffraction were employed to confirm the high quality of the as-synthesized ML-MoS_2_ (Supplementary Fig. S1).

**Figure 3:**
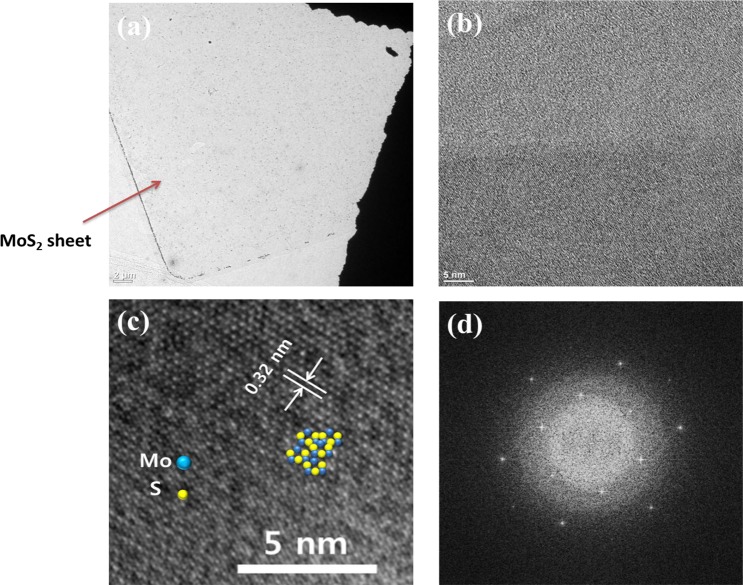
(**a**) Large-area uniformity for the synthesized MoS_2_ monolayer. (**b**) Typical TEM image of the as synthesized monolayer MoS_2_. (**c**) High-resolution TEM image of monolayer MoS_2_. The insets show modeled crystal structures for the MoS_2_ monolayer with the blue and yellow dots corresponding to Mo and S atoms, respectively. (**d**) SAED pattern for monolayer MoS_2_ confirmed the hexagonal structured phase.

The synthesized MoS_2_ films exhibit a very good crystalline quality as indicated by the Raman and PL measurements (Fig. 2c,d). To further elucidate the crystalline structure, we characterized the ML-MoS_2_ with a high-resolution transmission electron microscope (HRTEM). Figure 3(a) shows that the ML-MoS_2_ are uniformly continuous cross an area of micrometers. Figure 3(b) shows a typical TEM image of the as-synthesized ML-MoS_2_ that confirms uniformity with no defects such as cracks, which suggests a clean surface and high-quality for the produced ML-MoS_2_, in support of the Raman and PL results. The HRTEM image displayed in Fig. 3(c), together with the corresponding selected area electron diffraction (SAED) pattern in Fig. 3(d), reveal that the ML-MoS_2_ is a single crystal with a hexagonal lattice structure. A periodic honeycomb arrangement of the atoms with an inter-planar spacing of ∼0.32 nm is observed in Fig. 3(c). Mo and S atoms can also be identified in TEM images. The yellow and blue points correspond to the Mo and S atoms in the hexagonal structure, respectively.

**Figure 4:**
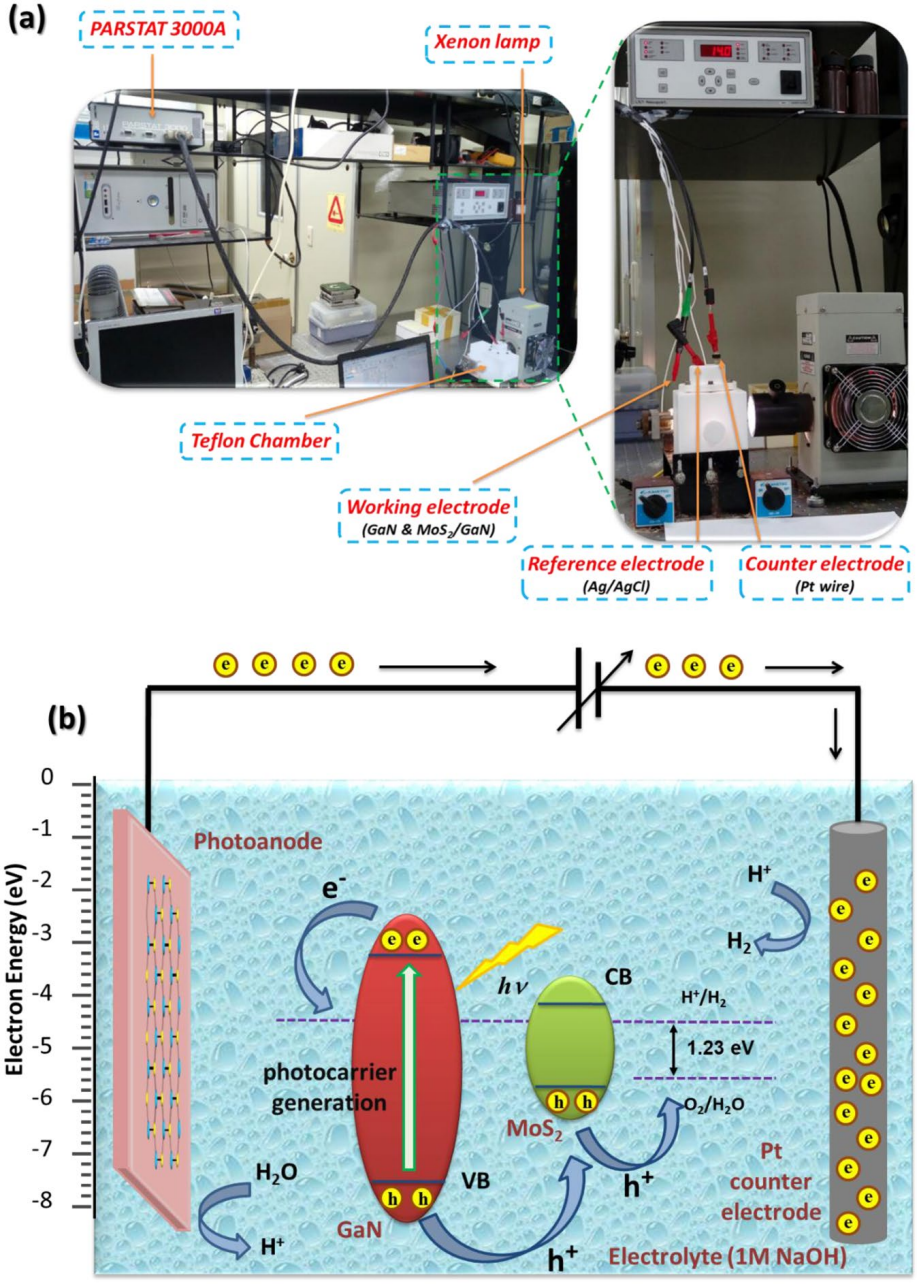
(**a**) Photographic images of the three-electrode home-made PEC cell used in the water splitting experiments. (**b**) Schematic diagram of the general PEC-WS process mechanism along with a band diagram for the MoS_2_/GaN photoanode with respect to the water redox levels.

To investigate the PEC properties of the ML-MoS_2_/GaN photoanode, a three-electrode home-made PEC cell was designed, as shown in Fig. 4(a). More details for the PEC measurements are provided in the Experimental section. MoS_2_/GaN served as the working photoelectrode to absorb illuminated light energy and to convert it into electron-hole pairs, which then started the respective water splitting half reactions. Due to the conventional band bending of the semiconductors, the photogenerated electron-hole pairs will be separated, where the holes will drift to the surface of the MoS_2_/GaN to strip oxygen from the water (water oxidation) and the liberated electrons will migrate to the counter electrode (Pt wire) to convert hydrogen ions into molecular hydrogen gas (hydrogen reduction), as illustrated in Fig. 4(b).

**Figure 5:**
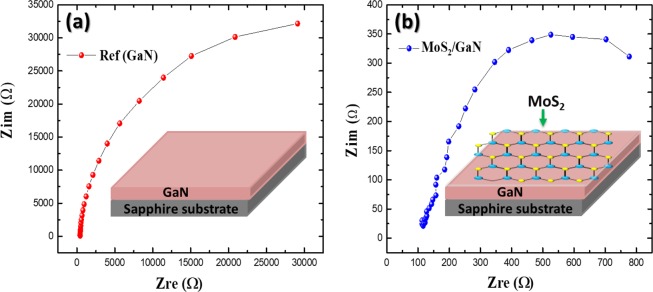
(**a**,**b**) Show the Nyquist plots for the GaN and MoS_2_/GaN photoanodes, respectively.

To understand the basic PEC parameters for the fabricated photoelectrodes, we carried out in-depth electrochemical investigations for both GaN and MoS_2_/GaN photoanodes using electrochemical impedance spectroscopy (EIS) under dark conditions. The EIS measurements were performed with the voltage amplitude of 20 mV for various frequencies (1.0 Hz–100 kHz). Figure 5(a,b) show the resulting Nyquist plots constructed from the electrochemical impedance spectra measured for GaN and MoS_2_/GaN photoanodes, respectively. Semicircles can be clearly distinguished in the Nyquist plots for each sample, which were used to understand the charge transfer process at the electrode/electrolyte interface, with the semicircle diameter being equivalent to the charge transfer resistance^51^. The smaller diameter of the semicircle for the MoS_2_/GaN electrode confirmed faster charge transfer process as well as more effective electron–hole pairs separation. Based on the results shown by Nyquist plots, we can conclude that the MoS_2_ plays a significant role in increasing the light absorption, accelerating the charge separation and transfer at the interface, which enhances the PEC performance. As a result, the MoS_2_/GaN heterostructure could be a potential photoanode for high-efficiency solar hydrogen generation because of fast charge separation and transport. To ensure that the MoS_2_/GaN photoanode hasn’t been degraded during the EIS measurement, a cyclic voltammetry (CV) scan is conducted before and after EIS measurement (Supplementary Fig. S2).

**Figure 6:**
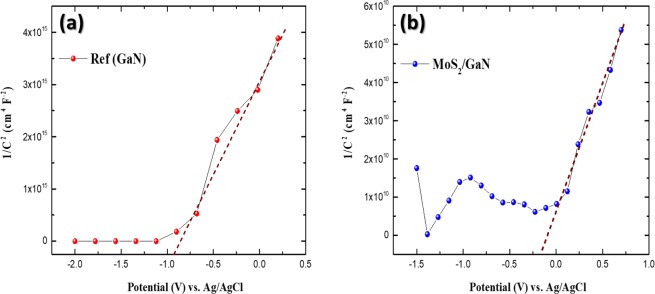
Mott−Schottky plots plotted as 1/C^2^ referenced to the Ag/AgCl electrode for (**a**) GaN and (**b**) MoS_2_/GaN photoanodes.

To gain further insights into the enhanced PEC performance of the MoS_2_/GaN photoanodes, capacitance–potential relation was investigated in 1.0 M NaOH electrolyte under dark conditions over the potential range of −1.5 to 0.7 V *vs* Ag/AgCl electrode. The space-charge capacitance of the semiconductor and flat-band potential values which can be estimated from Mott–Schottky (MS) plots, were analyzed according to the MS equation shown in Eq. (1)^52^

1 $$\frac{1}{{C}^{2}}=\frac{2}{\varepsilon {\varepsilon }_{0}e{N}_{A}}[(V-{V}_{fb})-\frac{{k}_{B}T}{e}]$$

where C is the depletion capacitance of the semiconductor layer, V is the applied potential, V_fb_ is the flat-band potential, N_A_ is the acceptor concentration, e is the elemental charge value, ε_0_ is permittivity of vacuum $({\varepsilon }_{0}=8.86\times {10}^{-12}F{m}^{-1})$, ε is the dielectric constant of the semiconductor, T is the absolute temperature, and k_B_ is the Boltzmann constant. The flat band potential can be obtained from the intercept of the plot tangents with the potential axis (C^−2^ = 0). The positive slopes of the tangent lines in the MS plot display the n-type property for both photoanodes, for which the lowest potential of the conduction band can be extremely close to its flat-band potential, as illustrated in Fig. 6(a,b). The flat-band potentials for the GaN and MoS_2_/GaN photoanodes were calculated to be −0.88 V and −0.21 V *vs* Ag/AgCl, respectively.

**Figure 7:**
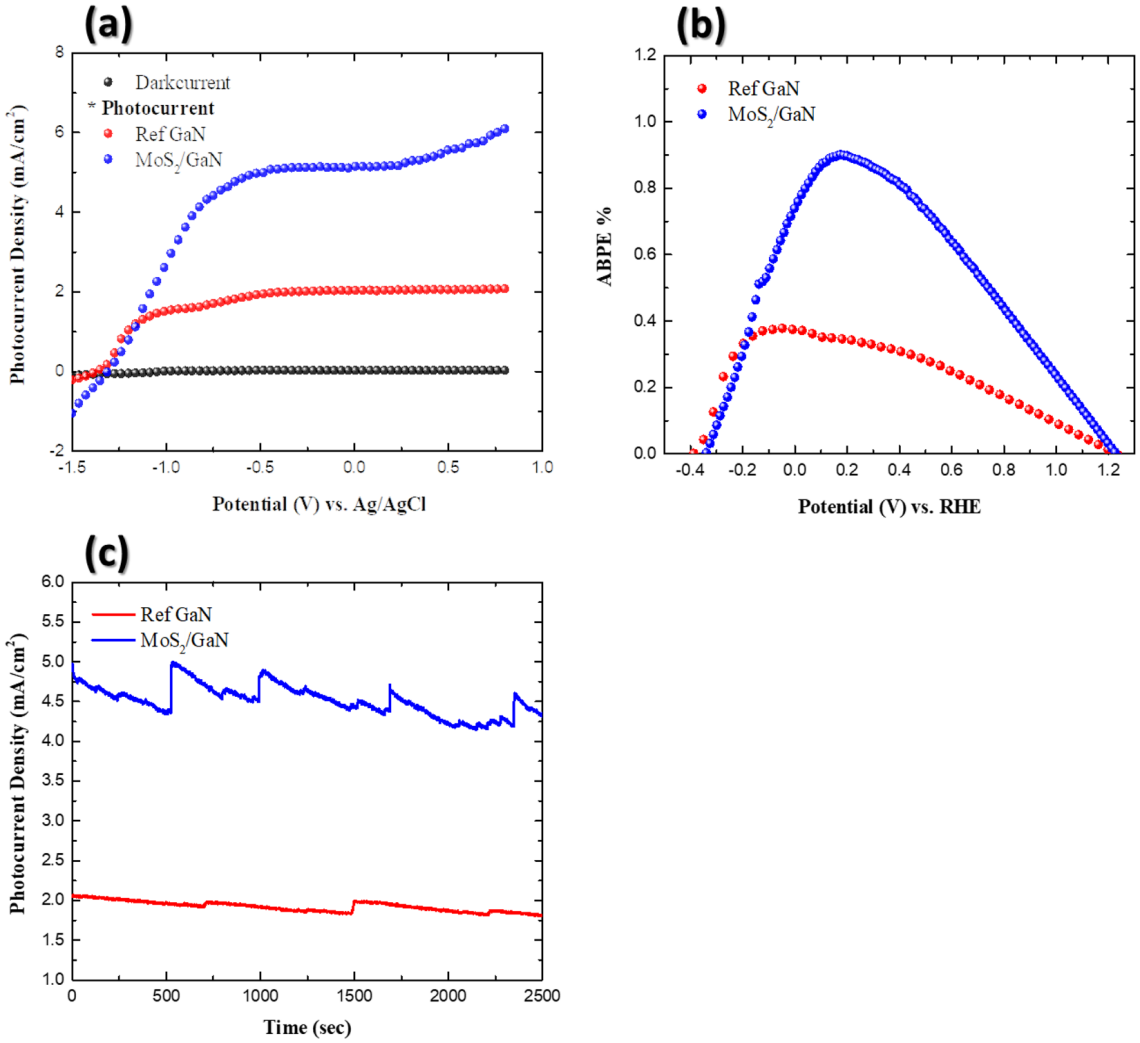
PEC performance of the MoS_2_/GaN photoanode as compared to GaN (**a**) LSV for the MoS_2_/GaN photoanode as a function of the applied potential referenced to the Ag/AgCl electrode. The data were obtained in the dark and under light illumination. (**b**) ABPE of the MoS_2_/GaN photoanode as a function of the applied potential referenced to RHE. (**c**) Chronoamperometric test of the MoS_2_/GaN photoanode as a function of working time measured at 0 V.

The PEC performance of the MoS_2_/GaN photoanode features light-driven water splitting process to investigate its potential as high-performance photoelectrodes. Linear sweeps voltammetry (LSV) were measured for pristine GaN and the MoS_2_/GaN photoanodes at a sweep rate of 20 mV/s, as depicted in Fig. 7(a). The photocurrent densities were measured as a function of the applied bias between the as-synthesized working electrodes (pristine GaN and MoS_2_/GaN) and the reference electrode, both in the dark and under illuminated conditions using 1 M NaOH as an electrolyte. Negligible dark currents were observed for all samples, revealing that the obtained photocurrent densities for all the prepared photoanodes are almost entirely generated through light irradiation. Compared to the pristine GaN, the MoS_2_/GaN photoanode showed increased photocurrent in the anodic bias condition. At zero bias *vs* Ag/AgCl, the MoS_2_/GaN photoanode exhibited a photocurrent density of 5.2 mA cm^**−2**^, about 2.6 times higher than that of the reference GaN. The observed photocurrent density is mainly subjected to the improved carrier extraction/collection efficiency and the faster charge carrier transport to the desired water redox interfaces. CV measurements for pristine GaN and MoS_2_/GaN photoanode including the Tafel plots obtained from CV curves have been conducted for further confirmation for the improved PEC properties (Supplementary Fig. S3). The superior water splitting behavior of the MoS_2_/GaN photoanode can be partly ascribed to the enhanced solar absorption due to the introduction of MoS_2_ as a cocatalyst with a narrow band gap (1.88 eV) that possesses the ability to absorb a significant portion of the entire solar spectrum, resulting in the generation of an increased number of electron-hole pairs^37,38^. In contrast, GaN with its large band gap energy will limit the solar absorption to photon energies in the UV region, which represents a small portion of the solar spectrum, thereby resulting in a small photocurrent density when exposed to the simulated sunlight^53,54^. The data in Fig. 7(b) shows the applied-bias-photon-to-current conversion efficiency (ABPE) to quantitatively evaluate the efficiency of the MoS_2_/GaN photoanode with respect to the applied potential. The ABPE is given according to Eq. (2)^55^

2 $$ABPE( \% )=100\times J(mA\,c{m}^{-2})(1.23-{V}_{app})/{P}_{light}(mW\,c{m}^{-2})$$

where $J$ is the current density under visible light irradiation, 1.23 V is the standard-state reversible potential of water, ${V}_{app}$ is the applied potential *versus* Ag/AgCl, and ${P}_{light}$ is power density of light illumination. The applied potential was converted relative to the reversible hydrogen electrode (RHE). The conversion from Ag/AgCl to RHE is calculated using the Nernst equation: ${E}_{(RHE)}={E}_{(Ag/AgCl)}^{0}+{E}_{(Ag/AgCl)}+0.059\times pH$, where $\,{E}_{(RHE)}$ is the converted potential relative to RHE, ${E}_{(Ag/AgCl)}^{0}$ is the standard potential of the Ag/AgCl reference electrode, which is equal (0.1976 V *versus* RHE at 25 °C), and ${E}_{(Ag/AgCl)}$ is the applied potential versus the Ag/AgCl reference electrode. The MoS_2_/GaN photoanode achieved an ABPE of 0.91% at about 0.21 V *vs*. RHE. This result is a significant enhancement compared to only 0.32% at 0 V *vs*. RHE for the reference GaN. Thus, the efficiency of the power conversion was increased by almost 3 times by using MoS_2_ as a cocatalyst coated onto GaN for the solar energy conversion device. To further investigate the photoresponse, long-term stability measurements were carried out using the chronoamperometry (CA) technique in 1 M NaOH under 500 mWcm^**−2**^ irradiation, with an applied bias of 0 V *vs*. Ag/AgCl. Figure 7(c) displays the CA curves versus time plots measured for 45 min. The recorded photocurrent densities maintain consistency with the values obtained from the J-V curves, so the photocurrents are stable without giving rise to photoinduced charging effect. The slopes of the curves reflect the stabilities of the photoelectrodes; a smaller slope indicates better stability. The MoS_2_/GaN photoanode exhibited a highly stable photocurrent density, approximately 5.2 mA cm^**−2**^ at zero bias *vs* Ag/AgCl, which represents a 2.6-fold enhancement compared to that of reference GaN. Measurements under successive illumination for 45 min reveals no obvious decay in the photocurrent, which is substantially improved compared to the previous research focusing on GaN-based photoanodes. In addition, SEM was carried out for MoS_2_/GaN after PEC stability measurements and showed that there is no degradation in the morphology (no corrosion) of the patterned ML MoS_2_ (Supplementary Fig. S4(a–f)). As a result, the patterned ML MoS_2_ inhibited the photocorrosion from the surface during the PEC experiments. X-ray photoelectron spectroscopy was performed after the stability test and it showed clear signal from MoS_2_ monolayer (Supplementary Fig. S5(a–f)). It is the evidence of stability of MoS_2_ during the long-term water splitting process. Furthermore, no degradation of the electrode material is observed by monitoring the surface morphology using an optical microscope following the PEC water splitting experiment, which suggests that there may not be any structural or morphological changes in the electrodes. This study clearly demonstrates that the MoS_2_ can significantly enhance the separation efficiency of photogenerated charge carriers in the MoS_2_/GaN photoanode.

## Conclusions

In summary, a facile strategy to synthesize a MoS_2_/GaN heterostructure photoanode for solar water splitting and hydrogen fuel generation was proposed. First, single-crystalline GaN was grown onto a c-plane sapphire substrate by MOCVD. Then, MoS_2_ was subsequently deposited by a Mo-sulfurization technique before being transferred onto GaN to form a MoS_2_/GaN heterojunction. The synthesized samples were analyzed in detail by TEM, PL, and Raman, and the fabrication of high-quality ML-MoS_2_ was confirmed. The resulting MoS_2_/GaN photoanode exhibited excellent PEC performance, including a photocurrent value of 5.2 mA cm^**−2**^ and a high photo-to-hydrogen conversion efficiency of 0.91%, which indicates great improvements in performance compared to pristine GaN. The significant enhancement in photocurrent density and efficiency that were observed can be attributed to the visible response of the MoS_2_ layer, which reduced the charge transfer resistance between the semiconductor and the electrolyte interface and the improvement of the charge separation in the MoS_2_/GaN heterostructure. This work demonstrates potential advantages of the MoS_2_/GaN heterostructure photoanode for PEC water splitting and provides an important methodology for the fabrication and design of III–V semiconductor surfaces with two-dimensional co-catalyst materials for solar-driven hydrogen production.

## Methods

### Synthesis of GaN on a c-plane sapphire substrate

Sample fabrication started with the growth of undoped GaN on a c-plane sapphire substrate by metal-organic chemical vapor deposition (MOCVD). Trimethylgallium (TMGa) and NH_3_ were used as precursors for gallium and nitrogen, respectively. To reduce the stress in the GaN thin film due to the lattice mismatch between GaN and sapphire, a very thin buffer layer of GaN was grown at low temperature of 600 °C. Then, the temperature of the reactor was increased up to 1175 °C for 2.5 μm-thick GaN. The flow rates of TMGa and NH_3_ were 292.67 µmol/min and 401.78 mmol/min, respectively, corresponding to a V/III ratio of 1372.9. The growth was performed for 3500 s at the reactor pressure of 100 torr while H_2_ was used as the main carrier gas.

### Synthesis of MoS_2_ on GaN

After lithographic patterning of the photoresist layer, a 1-nm-thick thin film of Mo was deposited onto a sapphire substrate by e-beam evaporation. The process began with the patterning of a Mo film array with rectangular windows ($110\times 190$μm^2^) by photolithography. Next, patterned Mo thin films deposited on sapphire substrates were placed in a quartz boat, which was in turn placed in the center of a chemical vapor deposition (CVD) tube furnace. A ceramic boat containing pure sulfur (≥99.99%, 0.8 g, Sigma-Aldrich) was placed in the upwind low-temperature zone in the quartz tube. During the reaction, the temperature in the low temperature zone was controlled to be just above the melting point of sulfur (113 °C). The quartz tube was first held under Ar at a flow rate of 100 sccm. The temperature was raised from room temperature to 750 °C at a ramp up rate of 75 °C min^−1^, and then held constant for 5 min before being naturally cooled down to room temperature in 60 min. To study solar-driven water splitting, CVD-grown single domains of MoS_2_ were transferred (using PMMA as a support film and 5 M NaOH/NaF solution) onto a GaN epilayer grown onto a sapphire substrate by MOCVD. The PMMA film was dissolved in acetone and the residue removed by annealing in Ar at 150 °C for 30 min.

### Material characterizations

The nanostructure and crystal quality of single domains of MoS_2_ were characterized by using high-resolution TEM with the atomic resolution, operating at 200 keV. Raman spectra for the MoS_2_ nanosheets were obtained by using a 532-nm laser excitation source operating at 6 mW. The room temperature PL for MoS_2_ was measured using a 532-nm laser with optical power of approximately 6 mW.

### PEC water splitting experiments

PEC experiments were performed in a three-electrode home-made cell wired to a typical potentiostat/galvanostat (Parstat 3000 A, Princeton Applied Research), operated by VersaStudio software. The as-synthesized samples were mounted onto a Teflon chamber cell having a size of $16\times 9\times 10\,c{m}^{3}\,(L\times W\times H)$. The MoS_2_/GaN photoanode, a Pt wire, and an Ag/AgCl electrode (sodium-chloride-saturated silver chloride electrode) were used as the working, counter, and reference electrodes, respectively. 1 M NaOH aqueous solution was used as the electrolyte. The MoS_2_/GaN photoanode was mounted onto the Teflon chamber, where the sample surface was directly in contact with the electrolyte through a hole with a diameter of approximately 0.5 cm. Then a conductive Cu wire was bonded to the MoS_2_/GaN photoanode surface using indium contact and wired to the counter electrode for hole transport. The sample was irradiated through a transparent quartz window using simulated sunlight provided by a 300 W Xe lamp (Newport 66902). The light irradiance was kept constant at 500 mW cm^−2^ during the measurements and illuminated surface area was about 0.5026 cm^2^. The chronoamperometry and linear scan voltammetry (LSV) (scan rate of 20 mV/s) experiments were performed using the single channel potentiostat. The performance of the MoS_2_/GaN photoanode was assessed based on the photocurrent density–voltage (J–V) curves evaluated both in the dark and under illumination.

## Supplementary information

Supplementary information.
