# Supplementary material for: Transferred monolayer MoS2 onto GaN for heterostructure photoanode: Toward stable and efficient photoelectrochemical water splitting
Source: Sci Rep. 2019 Dec 27;9:20141. doi: 10.1038/s41598-019-56807-y (PMC6934777; doi:10.1038/s41598-019-56807-y)
Supplement: Supplementary file 1 — Supplementary information. [file 41598_2019_56807_MOESM1_ESM.docx]

**Supplementary Information**

**Transferred monolayer MoS_2_ onto GaN for heterostructure photoanode: Toward stable and efficient photoelectrochemical water splitting**

**Mostafa Afifi Hassan^1^, Min-Woo Kim^2^, Muhammad Ali Johar^1^, Aadil Waseem^1^, Min-Ki Kwon^2^, and Sang-Wan Ryu^1,3*^**

^1^Department of Physics, Chonnam National University, Gwangju 61186, Republic of Korea

^2^Department of Photonic Engineering, Chosun University, Gwangju 61452, Republic of Korea

^3^ Optoelectronics Convergence Research Center, Chonnam National University, Gwangju 61186, Republic of Korea

* Corresponding authors

E-mail address: [sangwan@chonnam.ac.kr](mailto:sangwan@chonnam.ac.kr)

**Figure S1.** XRD pattern of MoS_2_/GaN photoanode.

**Figure S2.** Cyclic Voltammetry measurements for MoS_2_/GaN photoanode before and after electrochemical impedance measurements.


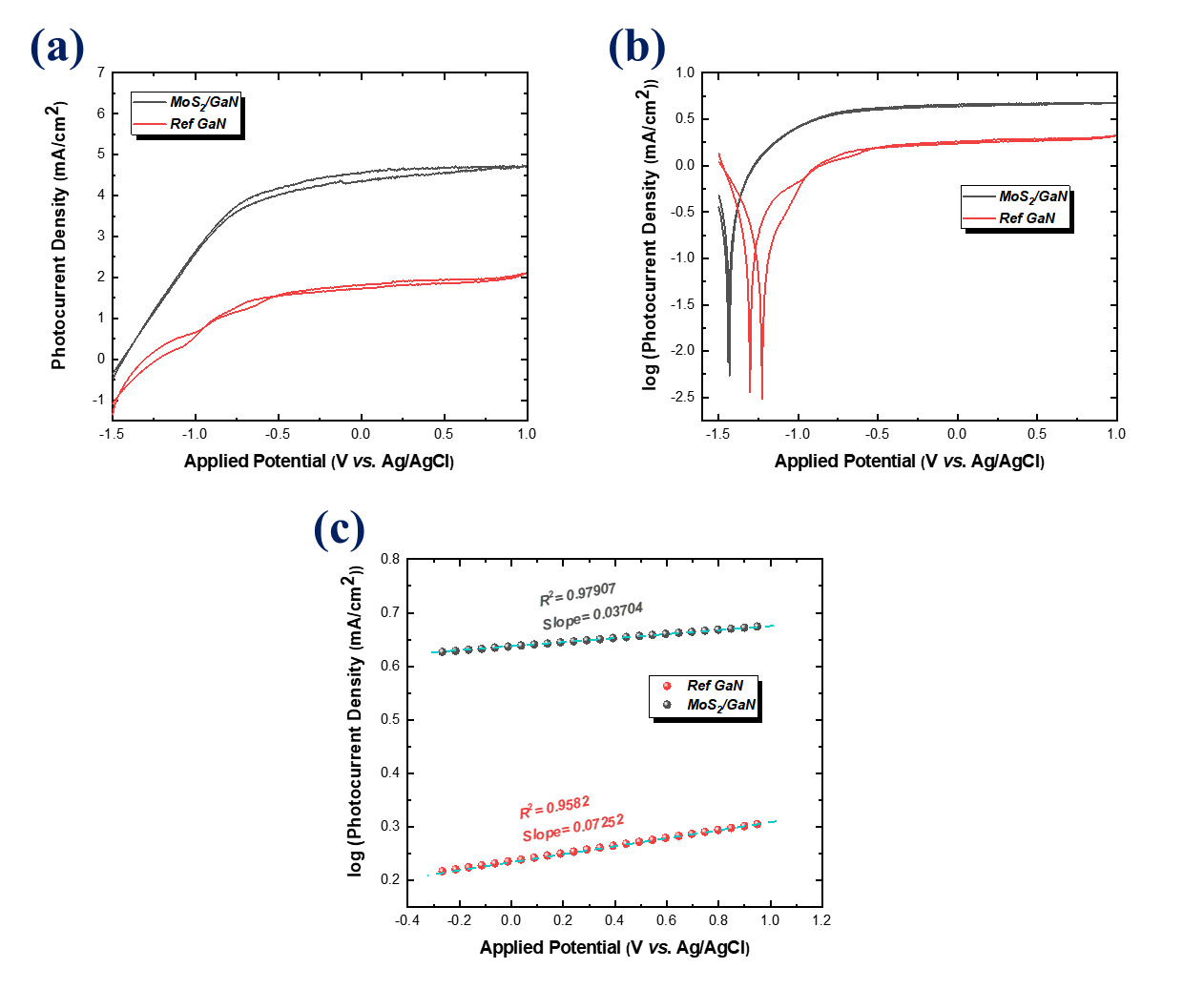


**Figure S3.** (a) Cyclic Voltammetry measurements for Ref GaN and MoS_2_/GaN photoanodes. (b) and (c) are Tafel plots constructed from Cyclic Voltammetry measurements for Ref GaN and MoS_2_/GaN photoanodes.


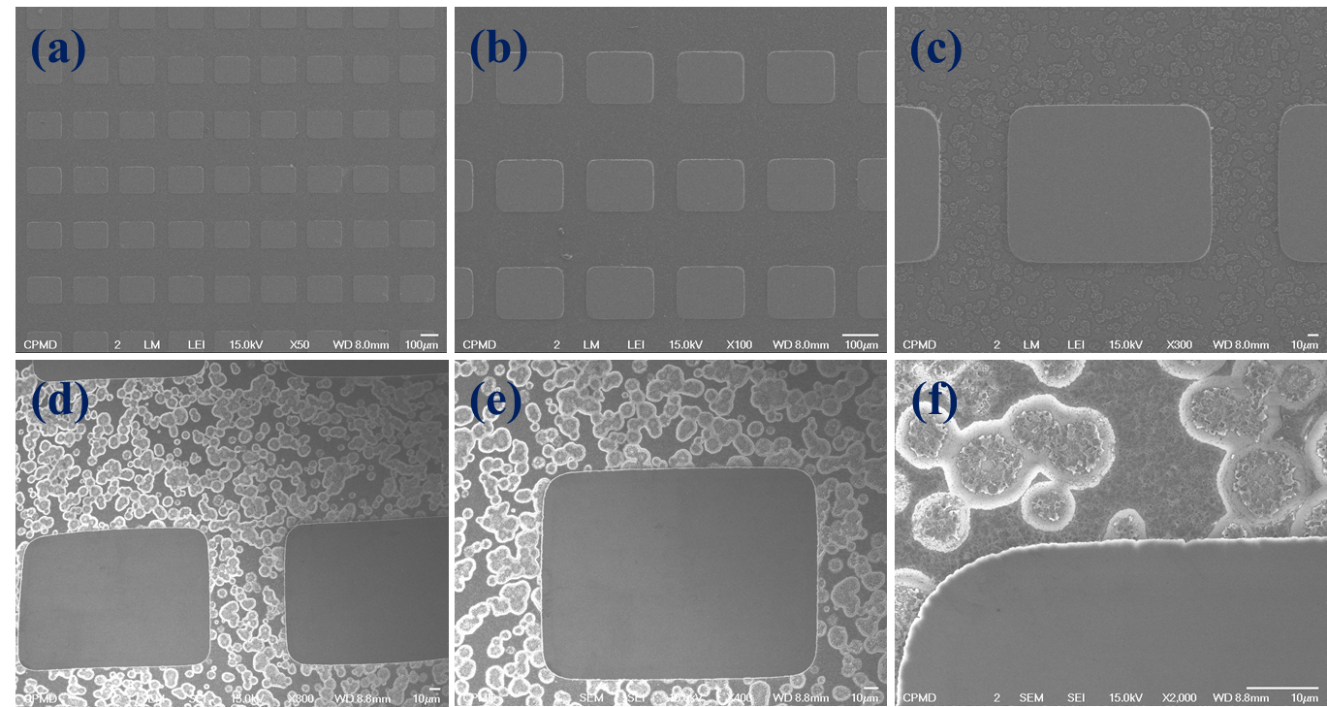


**Figure S4.** SEM images with different magnifications for MoS_2_/GaN photoanode after photoelectrochemical water splitting stability measurements.


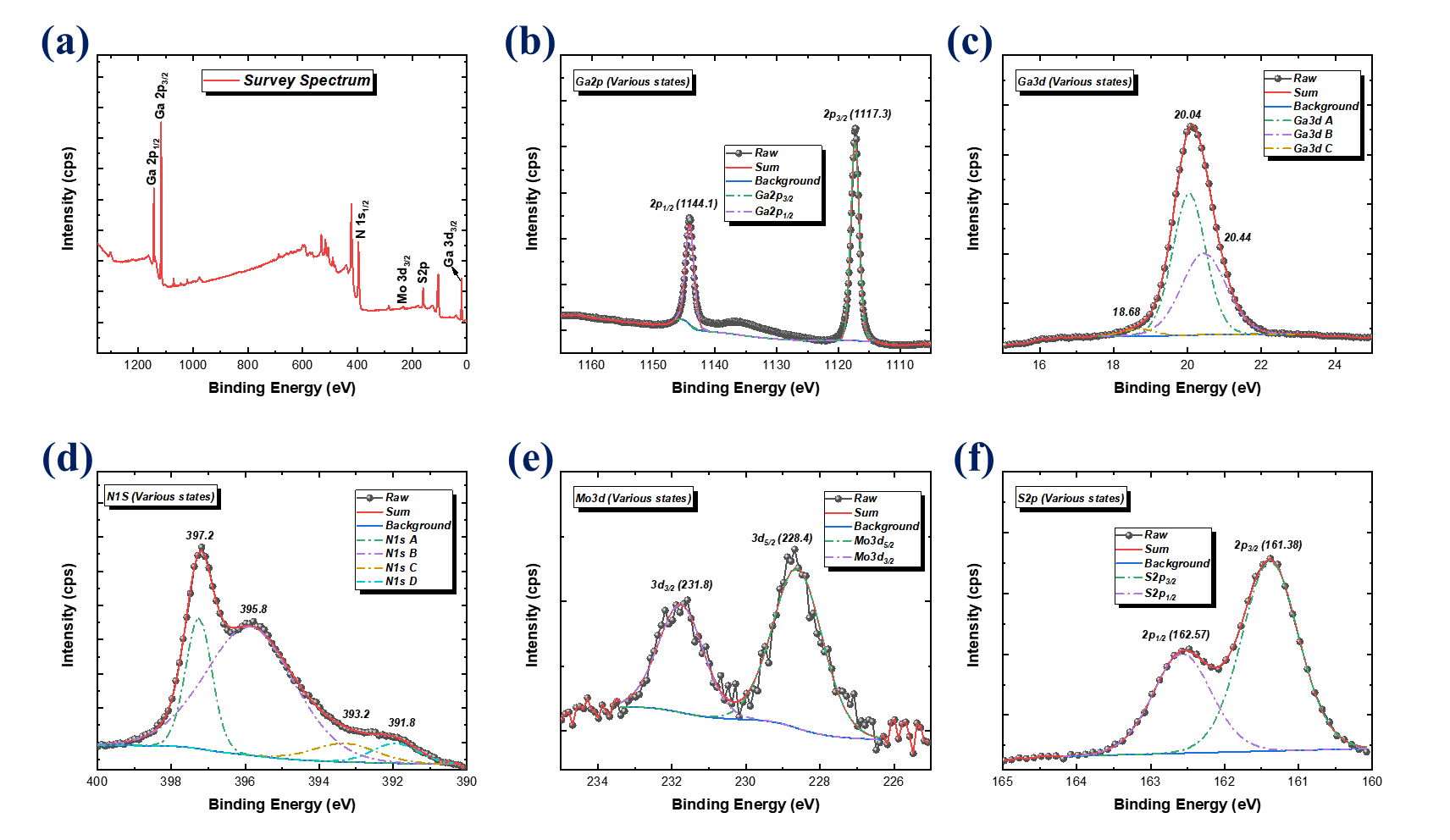


**Figure S5.** High-resolution XPS spectra of MoS_2_/GaN after PEC water splitting: (a) complete survey spectrum indicating the presence of Mo, S, Ga, and N in MoS_2_/GaN photoanode. HR-XPS of: (b) Ga 2*p*, (c) Ga 3d, (d) N 1s, (e) Mo 3d, and (f) S 2*p* regions.
